# Belowground plant allocation regulates rice methane emissions from degraded peat soils

**DOI:** 10.1038/s41598-024-64616-1

**Published:** 2024-06-25

**Authors:** Nijanthini Sriskandarajah, Chloé Wüst-Galley, Sandra Heller, Jens Leifeld, Tiia Määttä, Zutao Ouyang, Benjamin R. K. Runkle, Marcus Schiedung, Michael W. I. Schmidt, Shersingh Joseph Tumber-Dávila, Avni Malhotra

**Affiliations:** 1 Department of Geography, University of Zurich, 8057 Zurich, Switzerland; 2 Climate and Agriculture Group, Agroscope, Zurich, Switzerland; 3 College of Forestry, Wildlife and Environment, Auburn University, Auburn, AL 36849 USA; 4 Biological and Agricultural Engineering, University of Arkansas, Fayetteville, AR 72701 USA; 5 Department of Environmental Systems Science, ETH Zurich, 8092 Zurich, Switzerland; 6 Thünen Institute of Climate-Smart Agriculture, Bundesallee 68, 38116 Braunschweig, Germany; 7 Department of Environmental Studies, Dartmouth College, Hanover, NH 03755 USA; 8 Harvard Forest, Harvard University, Petersham, MA 01366 USA; 9 Biological Sciences Division, Pacific Northwest National Laboratory, Richland, WA 99852 USA

**Keywords:** Carbon cycle, Wetlands ecology, Ecosystem ecology

## Abstract

Carbon-rich peat soils have been drained and used extensively for agriculture throughout human history, leading to significant losses of their soil carbon. One solution for rewetting degraded peat is wet crop cultivation. Crops such as rice, which can grow in water-saturated conditions, could enable agricultural production to be maintained whilst reducing CO_2_ and N_2_O emissions from peat. However, wet rice cultivation can release considerable methane (CH_4_). Water table and soil management strategies may enhance rice yield and minimize CH_4_ emissions, but they also influence plant biomass allocation strategies. It remains unclear how water and soil management influences rice allocation strategies and how changing plant allocation and associated traits, particularly belowground, influence CH_4_-related processes. We examined belowground biomass (BGB), aboveground biomass (AGB), belowground:aboveground ratio (BGB:ABG), and a range of root traits (root length, root diameter, root volume, root area, and specific root length) under different soil and water treatments; and evaluated plant trait linkages to CH_4_. Rice (*Oryza sativa* L.) was grown for six months in field mesocosms under high (saturated) or low water table treatments, and in either degraded peat soil or degraded peat covered with mineral soil. We found that BGB and BGB:AGB were lowest in water saturated conditions where mineral soil had been added to the peat, and highest in low-water table peat soils. Furthermore, CH_4_ and BGB were positively related, with BGB explaining 60% of the variation in CH_4_ but only under low water table conditions. Our results suggest that a mix of low water table and mineral soil addition could minimize belowground plant allocation in rice, which could further lower CH_4_ likely because root-derived carbon is a key substrate for methanogenesis. Minimizing root allocation, in conjunction with water and soil management, could be explored as a strategy for lowering CH_4_ emissions from wet rice cultivation in degraded peatlands.

## Introduction

Over the Holocene, peatlands have accumulated 30% of the world’s soil organic carbon (SOC) while covering only 3% of the land area^1–3^. However, peatlands have been extensively drained for agricultural uses^4^, leading to considerable CO_2_ emissions^5^. One solution for rewetting drained peatlands while maintaining their agricultural utility, is wet crop cultivation, for example wet rice cultivation^6,7^. However, rice cultivation, currently accounting for ~ 20% of total agricultural CH_4_ emissions globally^8–10^, would lead to increased CH_4_ emissions. Water table management and the addition of mineral soil are two strategies by which rice CH_4_ emissions might be reduced^11^. For example, mid-season drainage for a period of typically 1–2 weeks is known to reduce CH_4_ emissions, both during and following the drainage period, whilst maintaining yields^11^. The addition of mineral soils to peat soils is also a strategy increasingly used by farmers to ease the management of poorly-drained soils (e.g., to improve flood response or to be able to operate heavy machinery on the soils)^12^. Previous work has shown that lower water tables and mineral soil addition can decrease CH_4_ emissions in rice grown on degraded peat soil^13^. However, it remains unclear how plant allocation strategies and properties (traits) respond to soil and water management^14^ and, in turn, how plant (especially root) traits influence CH_4_^15^.

Water and soil treatments could influence plant allocation and traits in a variety of ways that would influence CH_4_. For example, water stress could increase plant carbon allocation to roots^14,16^. Soil treatments could influence root growth via changes in macro and micronutrients, soil pore structure, and soil water holding capacity. A reduction in plant-available macro or micronutrients could trigger increased below-ground allocation^17,18^. In turn, altered allocation belowground as well as changed root traits could have a range of confounding effects on net CH_4_ flux^19^. First, roots act as conduits through which CH_4_ is produced in the deeper layers of soil and can be transported to the atmosphere^20,21^. Thus, an increase in root allocation could increase CH_4_ transport and enhance the CH_4_ flux. Second, through these same conduits, oxygen may be transported into the saturated soil layers where CH_4_ oxidation may occur thereby reducing the net CH_4_ flux^20,22^. Third, root exudates may fuel heterotrophic microbes, leading to more CH_4_ production^23–25^ or, in contrast, consuming more CH_4_^26^. It remains unclear which plant traits are influenced by water and soil management^27^, and subsequently, how root traits and CH_4_ fluxes are related^19^.

To address research gaps on plant and particularly root trait response to water and soil amendments, and downstream effects on CH_4_ release from rice, we leveraged an existing mesocosm rice experiment in Switzerland. The experimental plots contain two water table conditions (low vs high) and two soil types (degraded peat, and degraded peat covered with mineral soil)^13^. Previous work from this experiment suggests that a lower water table and mineral soil cover can greatly decrease CH_4_ emissions from paddy rice^13^, but the role of root traits and biomass allocation in driving this reduction remain unanswered. Thus, here, we address the following research questions: (1) How do water table and soil amendments influence rice below:aboveground allocation strategies and root traits (biomass, length, diameter, root tissue chemistry)? (2) How do plant allocation and trait changes influence CH4 fluxes?

First, we hypothesize that under a lower water table, rice plants will allocate a greater fraction of biomass belowground (relative to aboveground) to increase water uptake under water stressed conditions. Simultaneously, root traits related to water/nutrient uptake capacity such as specific root length (SRL) and root surface area, will also increase. Second, we hypothesize that the peat-only soil will generate more above and belowground biomass relative to the mineral-covered peat. We expect this increased productivity because, in this experiment, the degraded peat soil has more plant-available nutrients than peat with mineral cover soil. In mineral-covered peat, plants likely allocate more biomass belowground than aboveground compared to peat-only, to compensate for nitrogen limitation. Under this nutrient limitation, we also expect increased resource acquisition traits such as SRL and root surface area, in the mineral soil. Lastly, we hypothesize that plants with high belowground biomass and larger root diameter will emit more CH_4_ due to both high substrate availability from root exudates and greater CH_4_ transport through their thicker roots^19,28^. Our study contributes to a better understanding of how water table and soil management can influence allocation strategies in rice and how these plant traits are related to CH_4_ emissions.

## Materials and methods

### Experimental site and design

**Figure 1:**
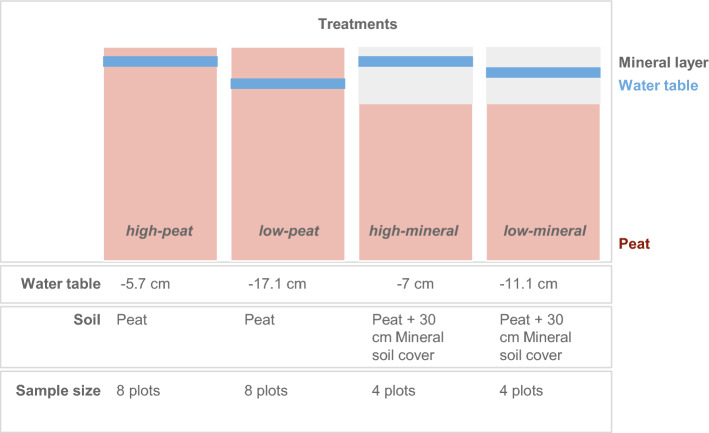
A schematic of the four types of treatments (high-peat, low-peat, high-mineral, low-mineral) evaluated in this study. Experimental plots had high (saturated) or low water treatments and degraded peat-only or degraded peat covered with ~ 30 cm mineral soil treatments. The average growing season water table height and sample sizes are also shown (note the unbalanced design).

**Figure 2:**
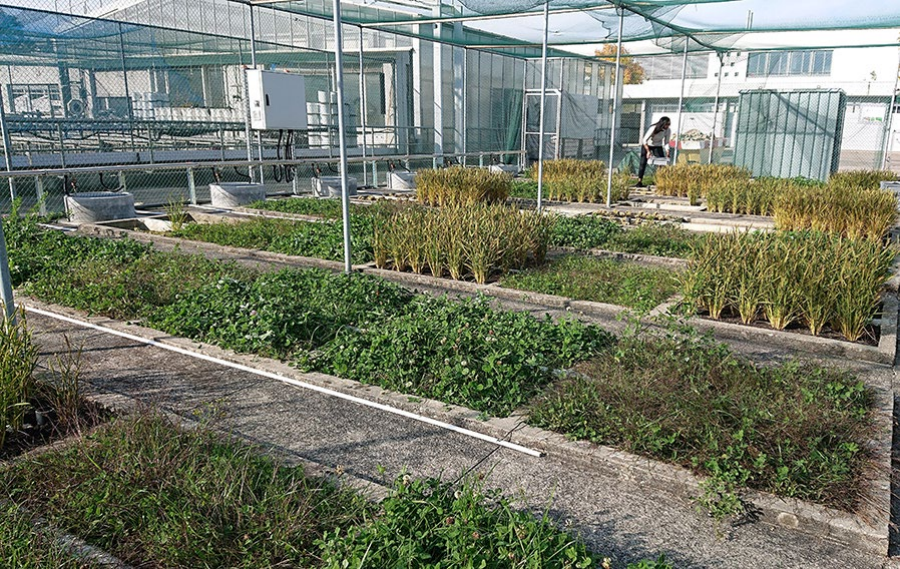
Image of the Agroscope rice experiment, with 1.2 × 1.2 m growth plots showing grass and rice plots (Taken on October 18, 2021).

The Agroscope rice mesocosm experiment (Figs. 1 and 2) is located in Zurich Affoltern, Switzerland (47° 25.8′  N 8°  31.2′  E with an elevation of 466 m.a.s.l.). The local climate is characterized by a mean annual temperature of 9.82 °C and a mean annual precipitation of 1026 mm (from 1990 to 2021)^29^. This experiment was conducted between March and October 2021 (See Table S1 for dates and details of the experiment). Rice was grown in 24 experimental plots of 1.2 m × 1.2 m size and 1.4 m depth. The 24 plots had different soil (with or without a 30-cm mineral soil layer) and water treatments (targeting high at − 6 cm or low at − 20 cm) combinations (Fig. 1). Note that water table targets differed in achieved water tables due to the different subsidence of the different soils (detailed below). In the end, the four treatments were: peat and high water table, peat and low water table, peat plus mineral soil cover and high water table, and peat plus mineral soil cover and low water table (hereafter, high-peat, low-peat, high-mineral, low-mineral; Fig. 1).

### Soil treatments

The plots (1.4 m deep) were filled with either just degraded peat (16 plots) or degraded peat with a 30 cm mineral soil layer on top (8 plots; Fig. 1). We added a 30 cm mineral layer because this is the typical layer thickness added in Switzerland, where this practice is carried out^12^. This resulted in an addition of ~ 388 kg of mineral soil cover treatment (hereafter, ‘mineral’).

The degraded peat soil was taken from an 80 year old agricultural site in Affoltern am Albis (47° 16′ N, 8° 27′ E). This degraded peat soil had an organic carbon content (C_org_) of 27.4%, C:N ratio by mass of 19.9 and a soil pH of 6.0^13^ and was well mixed before adding to our plots, thus the vertical characteristics of a peat soil are not represented in the experiment. The mineral soil covering the degraded peat soil was taken from a farm in Rüthi, St. Gallen (47° 18′ N, 9° 32′ E) and was a loam containing 41.4% sand, 12.3% clay and 46.3% silt^13^. This soil material is calcareous and has the characteristics: C_org_ 0.6%, C:N ratio of 12.6 and a pH of 7.6^13^. Before application, the mineral soil was mixed with compost (10 kg [dry matter] per plot, pH 8.1, C_org_ 20.5%, C:N = 13.2), a measure also used by Swiss farmers^12^. Total C and N content were analyzed with a CHNS–O elemental analyser EuroEA3000 (HEKAtech, Germany). It is worth noting that pH in the degraded peat plus mineral soil is higher than that of a typical unmanaged peatland, where pH is usually lower than 6^30^. Thus our experimental mineral soils likely have much higher decomposition rates than a typical unmanaged peatland.

### Water table treatments

Across the 24 plots, 12 had a ‘low’ and 12 had a ‘high’ water table (Fig. 1) but due to differential subsidence, the water table was also dependent on soil treatment. The ‘high’ water table plots had a growing season water table depth of − 6 and − 7 cm (below the surface), without and with mineral cover, respectively^13^. The ‘low’ had growing season water table depths of − 11 cm with mineral cover; and − 17 cm without mineral cover (Fig. 2). With minor exceptions (see below), the water levels were maintained at these depths throughout the vegetation season and unwanted variations were adjusted (for example after heavy rains; Figure S1 explains the experimental infrastructure). In the high water table, mid-season drainage was applied between 4 and 12th August, where the water level was lowered to − 100 cm. Additionally, the water level was lowered (to ca. − 10 cm) the day seedlings were planted and hours prior to fertilization. The water level of all plots was reduced to − 100 cm the week prior to harvesting. The water level was calculated based on data from water table loggers, adjusted for soil subsidence (Figure S1). The soil subsidence was measured once every month during the vegetation period.

The rationale for the two water table depths is as follows: the high water table roughly corresponds to the − 5 cm optimum water table depth for minimizing greenhouse gas emissions^31^. The low water table corresponds to a depth at which conventional management (in the Swiss water management context) can take place and therefore at which farmers might be able to cultivate rice without having to adjust management practices too much.

As a result of the water and soil treatments, resulting growing season average volumetric water contents varied and were as follows for the different treatments: high-peat = 0.65 m^3^ m^−3^ (excluding the mid-season drainage), low-peat = 0.63 m^3^ m^−3^, low-mineral = 0.42 m^3^ m^−3^ high-mineral = no moisture data from these plots (as no CH_4_ was measured). Averages are from half-hourly soil moisture measurements from mid-July to October, using Teros-11^®^ (METER Group) soil sensors at 5 cm depth^13^.

### Rice cultivation

The experiment used the rice variety, ‘Loto’ (*Oryza sativa* L*.*), typically grown as paddy rice in the cool temperate moist climate of the central plateau of Switzerland^32,33^. Rice seeds were sown in seed trays in commercially-available sowing compost. The seed trays were placed for 4 weeks in climate chambers and then for 1 week into a greenhouse. On the 26th May 2021, at the three-leaf stage, the seedlings were transplanted to the experimental site (Fig. 2). Each plot was planted with 34 plants^13^, resulting in a density of 24 plants m^−2^. The rice plants were fertilized as seedlings with Wuxal(R) (Syngenta Agro AG), an NPK mineral fertilizer with micronutrients (K, B, Cu, Fe, Mn, Mo, Zn), and after planting out (three times) with an ammonium nitrate fertilizer (NH_4_NO_3_ with Mg and S), P_2_O_5_ and K_2_O mineral fertilizers (Table S1)^13^. The fertilizer amounts correspond to common greenhouse practice and the Swiss fertilizer recommendations^34^.

### Plant sampling and processing

To characterize root traits, we collected one random rice plant per plot after harvest (18 Oct. 2021). Thus, a total of 24 plants were sampled, i.e. 8 × peat with high water table, 8 × peat with low water table, 4 × mineral cover with high water table, and 4 × mineral cover with low water table (Fig. 1). The rice plant was separated into above- and belowground components. The aboveground part, which included leaves, stem, and rice, was dried at 60 °C for 3–4 days and weighed. The belowground part, including roots and soil, was collected, and immediately brought to the lab. The soil sample including the roots was circa 16 cm long × 16 cm wide × 18 cm deep. In the lab, the soil was washed using distilled water in an ultrasonic bath for 2–8 h to remove the soil from the roots by ultrasound induced cavitation forces. These partially clean root systems were then stored frozen at − 20 °C to prevent decomposition of the roots until further processing.

### Laboratory analysis

Each root system was thawed for 24 h in the fridge for further cleaning and processing. Once thawed, following standard methods^35^, roots were washed again but this time more thoroughly and until entirely free of soil. This was done using magnifying glasses, distilled water, forceps, and paint brushes to clean the remaining soil from the root surface. After cleaning, the roots were scanned in a scanning tray (29.7 cm × 42.0 cm) using a Canon Image Runner Advance C5535i with the integrated scanner in grayscale mode at 600 DPI (dots per inch). The scans were then saved as a TIFF-file for further image analysis. After the scanning, the wet weight of the roots was measured, and the roots were dried at 60° for 1–2 days. Subsequently, the roots were weighed again to determine their dry weight. The sampled soils from which the roots were processed had slightly different volumes. Thus, the dry root biomass was normalized to a soil volume of 15 × 15 × 15 cm (3375 cm^3^) by dividing root biomass weight with the collected soil volume and multiplying by 3375 cm^3^.

### Image processing root scans to quantify root traits

The root scans were analyzed using Rhizovision Explorer v2.0.3^36^, an open-source software developed for root image processing. For each of the 24 root systems, we were able to obtain the following root traits from Rhizovision: total root length, total root surface area, total root volume, root average diameter and root length for diameter bins from 1 to 6 mm (see Figure S2 and the supplementary section on “Root trait quantification using Rhizovision software”). We also calculated specific root length (SRL) as the ratio of the total root length to BGB, which provides an indication of root length investment per unit mass and is expected to increase when a plant is resource stressed^37,38^.

### Methane and ancillary data

Methane fluxes and aboveground biomass (hereafter, AGB) were measured in the high-peat, low-peat and low-mineral treatments in a previous study^13^. These treatment names correspond to the following treatment names from the previous study^13^: RH, RM, and RM + min, respectively. Briefly, the CH_4_ fluxes were measured twice a week during the growing season, using a manual dark static chamber attached to a gas analyzer (cavity ring-down spectrometer; model G2308, Picarro Inc., Santa Clara, CA, USA). The chamber enclosure time for each individual plot measurement was 15 min. The first CH_4_ flux measurements were conducted six days prior to transplanting the rice seedlings and continued until the time of harvesting the last rice plants. Additional gas measurements were carried out immediately prior to, and following, fertilization and changes in the water table (e.g. mid-season drainage). For the present study, the average value of the 44 total measurements that were taken for each plot was used to represent CH_4_ emissions. In our analysis, CH_4_ fluxes were measured from 12 of the 24 root plots (4 × high-peat, 4 × low-peat, and 4 × low-mineral plots). Following the micrometeorological sign convention, positive CH_4_ fluxes in this study are referred to as CH_4_ emissions to the atmosphere (source), and negative fluxes as CH_4_ uptake by the soil (sink).

### Statistical analysis

We used the following variables in our analyses: AGB, BGB, total biomass (AGB + BGB), the ratio of belowground and aboveground biomass (BGB:AGB) and root traits (total root length, total root averaged diameter, total root surface area, and total root volume) from image analysis. We also used growing season average CH_4_ fluxes from a previous study^13^. Ancillary variables included mean and standard error of water table depth, and soil carbon, nitrogen, oxygen and carbon:nitrogen ratio^13^. All statistical analyses and data visualizations were performed using JMP^®^, Version 15.2.0 (SAS Institute Inc., Cary, NC, 1989–2021).

Due to our plant trait data being unbalanced, non-normal and small in sample size, we used a non-parametric Kruskal–Wallis test to evaluate the treatment effects on plant traits and additionally compared treatments using the Steel–Dwass method for pairwise comparisons. The goal of these statistical tests was to compare BGB, AGB, total biomass, and BGB:AGB among the different soil and water treatments (hypothesis I and II). For our third hypothesis, we first used multiple linear regression to establish soil, water and plant trait predictors of CH_4_ fluxes. We then also used bivariate linear regressions to further investigate relationships between CH_4_ and key plant-trait predictors. We log-transformed CH_4_ data to meet the assumption of normality for linear regressions. Lastly, to explore the trait covariation and the relationship between CH_4_ and traits, we also used principal components analyses (PCA).

## Results

### Water table and soil type influence rice biomass

**Figure 3:**
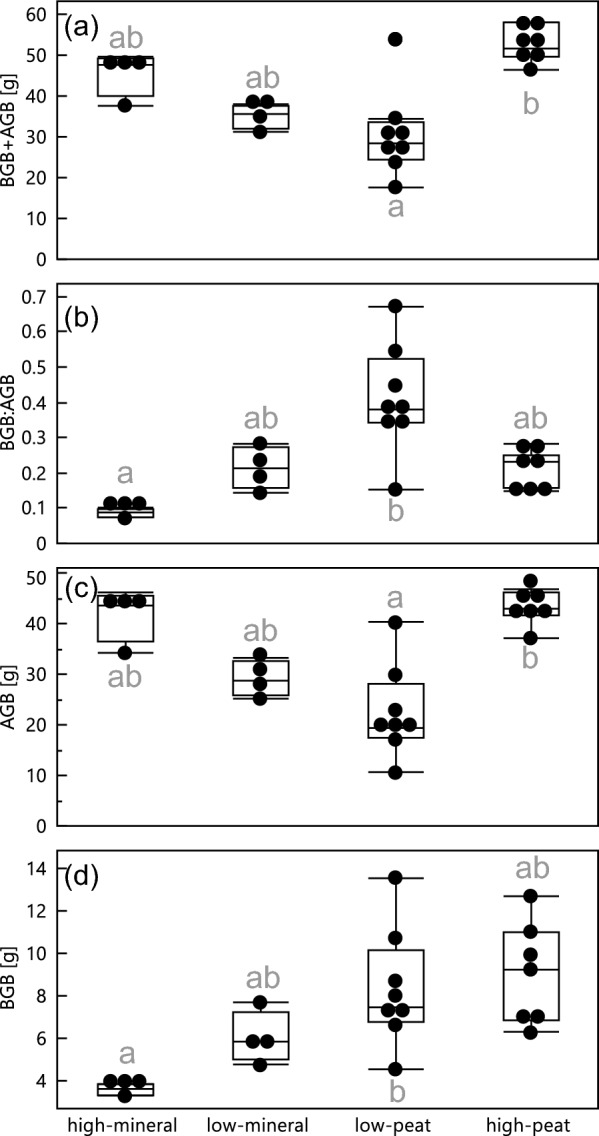
The response of above (AGB) and belowground (BGB) biomass, BGB:AGB and total biomass (AGB + BGB) to high or low water table and peat or peat with mineral soil treatments (referred to as mineral in the figure). For each panel, treatments that do not share a letter code denote pairwise significant differences.

The water table and soil treatments significantly affected rice BGB and AGB and supported our hypothesis that a lower water table increases plant BGB allocation (Fig. 3b). However, our hypothesis that nutrient-poor mineral soils will have a higher belowground allocation than peat soils was not supported. We observed considerable differences in AGB and total biomass between water table treatments in peat soils, and in BGB and BGB:AGB between the high-mineral and low-peat treatments (Fig. 3; Table S2). Low water tables in peat soil approximately halved both above and total biomass relative to high water tables. With lower water tables, AGB decreased from 43 to 19 g (median values in Fig. 3c) and total biomass (AGB + BGB) from 51 to 28 g (Fig. 3a). Meanwhile, BGB halved in the high-mineral, compared to the low-peat treatment (7.5 to 3.6 g shift in median values; Fig. 3d), leading to a 4 × reduction in BGB:ABG allocation (from 0.4 to 0.1; Fig. 3b).

### Treatment response of other root traits

**Figure 4:**
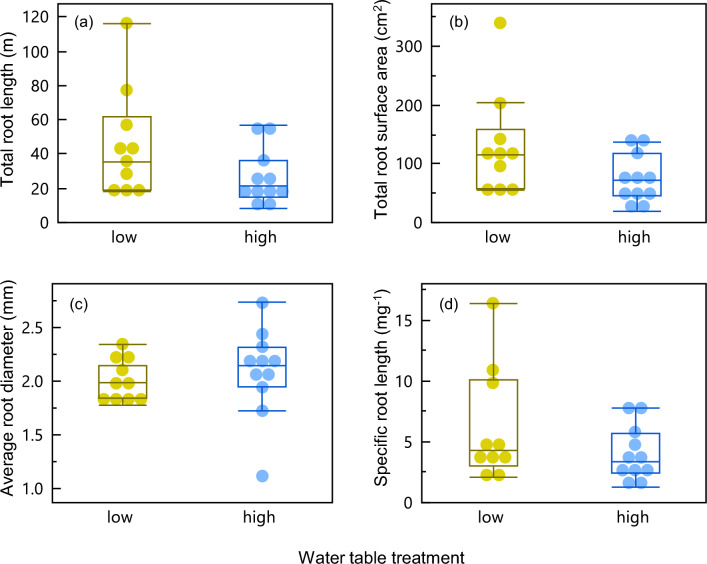
Root traits plotted against water tables. Specific root length (SRL) was computed as root length divided by belowground biomass (BGB). None of the root traits showed significant differences between water or soil treatment.

Root traits except BGB and BGB:AGB showed very little variation between peat and mineral soil types (Table S3). Across water treatments, no significant difference was seen in the root traits either, but some interesting trends were present (Fig. 4). Notably, roots were slightly but insignificantly longer per unit dry mass (SRL, Fig. 4) under water-stressed conditions. Although root traits varied considerably, the low water table had higher medians and interquartile ranges for all root traits except diameter (Fig. 4; Table S3). We also noted that the low-peat treatment had a much higher total root length when compared with other treatments and especially when compared to the high-mineral treatment (Table S3). Though not significant at p < 0.05, it is worth noting that high-mineral treatment had roughly half the total root length of the low-peat treatment (Kruskal–Wallis test chi square = 6.2, p = 0.0980; Steel–Dwass pairwise comparison Z = 2.17 p = 0.13). While root traits did not show significant treatment effects, we still report trait values and trait covariation as these are important baseline values for rice plants (Table S3).

### Root biomass and CH_4_ emission positively correlated in low water table

Net flux average values of CH_4_ ranged from 2.4 g CH_4_ m^−2^ y^−1^ in the low water table treatments to 6.4 g CH_4_ m^−2^ y^−1^ in the high water treatments^13^. A multiple regression model that included BGB, soil treatment, water table treatment and the interaction between BGB and water table treatment explained 78% of the variation in CH_4_, where only an interaction term between BGB and water table level was significant (model output reported in Table 1; See Figure S4 for non log transformed CH_4_ values).

**Table 1:** Best fit model (after removing other root traits and interactions between traits and soil treatment) of CH_4_ emission (log mg CH_4_ m^−2^ growing season^−1^).

Model term	Estimate	Std error	t ratio	p value
Intercept	9.02	0.64	14.12	0.0000
BGB	− 0.04	0.08	− 0.59	0.5756
Soil treatment [mineral]	− 0.19	0.19	− 1.01	0.3532
Water table treatment [low]	− 0.33	0.19	− 1.71	0.139
(BGB − 7.9)*Water table treatment [low]	0.25	0.08	3.25	0.0174

Model R^2^ = 0.78, p value = 0.0375, n = 12. Note that the model was run on log transformed values of CH_4_ flux to resolve issues of non normality.

**Figure 5:**
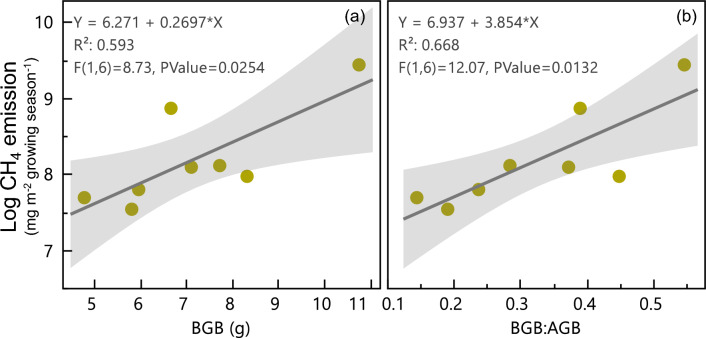
Belowground biomass, (BGB) and the belowground:aboveground (BGB:AGB) as predictors of CH_4_ flux. Only the low-peat and low-mineral treatments are considered in this analysis.

Given this interaction between BGB and low water tables, we analyzed water treatment individually. We found that in the low water table treatments, BGB and BGB:AGB were significantly positively related to CH_4_ flux (Fig. 5). In the high water table plots, we did not have enough data (n = 4) to establish a relationship between CH_4_ flux and BGB (Figure S4).

## Discussion

In this study, we evaluated the response of plant biomass allocation and root traits to different soil and moisture conditions in an experimental rice (*Oryza sativa L.*) system and investigated root trait linkages to CH_4_ flux. We found that plants allocated the least belowground biomass in water-saturated and mineral-covered peat soils and the most in water-stressed peat soils. Among our measured plant traits, BGB and BGB:AGB allocation had the strongest link to CH_4_ flux but only in low water table conditions. Thus, our results reveal that rice response to different soil–water treatments differs between above and belowground plant organs and that the belowground trait response is a predictor of CH_4_ dynamics.

### More total biomass with higher water table levels but variable belowground allocation

In our study, as expected for rice, higher water table levels in general increased total plant biomass^9,39^ and lower water tables increased plant allocation to roots^40^. Water-stressed plants in the low water treatments are expected to allocate more BGB to increase their water uptake capacity^16,41–44^. We also saw some support for this in the other root traits wherein roots from low water table conditions had higher SRL and length allocation (Table S3). We observed a decrease in AGB with lower water tables, which could be due to water limitation for the plant, given the high water demand of rice plants^45,46^. While the AGB response to water availability is often observed in rice, our study provides evidence that BGB:AGB also responds to the water table changes as seen in other ecosystems and in intact peatlands^47,48^. A further explanation for lower AGB under low water tables could be due to plant stress from temperature variations^49^. Under high water tables, temperature variations (e.g., low night-time temperatures) would be buffered by the water but low water tables would likely see stronger variations in soil temperatures that could negatively influence plant growth^50,51^.

### Decreased BGB with mineral soil additions

We hypothesized that the mineral soil treatments would have greater BGB than the peat soil due to low nutrients in the mineral soil (Figure S3 and Table S4). We observed the opposite wherein the mineral soil treatment had the lowest BGB and belowground allocation (Fig. 3b and d). One reason for this could have been that the plants in the mineral soil treatment were overall nutrient limited. However, AGB and total biomass data do not support this reason because AGB is not low in mineral soil treatments; rather it is lowest in the peat-only soils (Fig. 3c).

We did not have detailed post-experiment data on macro and micro nutrients to fully assess the role of nutrient limitation in our results. However, we have post-experiment soil carbon (C) and nitrogen content (N), and C:N that suggests that N availability was much higher in the peat soil; Figure S3). Even though C:N has been shown to be a sensitive parameter for peatland degradation^52^, our C:N data could reflect nutrient use that occurred during the experiment, rather than nutrient status and availability itself. We also have nutrient data from pre-experiment soils, where we find some support for different nutrient availability in the mineral and peat soils (Table S4). Notably, cation exchange capacity was almost 20 times higher in the degraded peat than in the mineral soil suggesting potentially higher nutrient retention in the peat (Table S4). Calcium, magnesium and sodium were also higher in the peat than in the mineral soil. However, potassium was higher in mineral soils compared to peat soils. Potassium is generally considered more important for plant growth than our other reported nutrients^53–55^ and may explain why we do not see high root allocation in the mineral soil treatment. Furthermore, mineral soil mixed with peat has been shown to increase phosphate, potassium, iron and magnesium availability^56^ and higher N retention^57^. Such retention effects could therefore have influenced the availability of nutrients derived from the fertilization and compost that was added to our mineral soil treatment. Furthermore, the lack of high BGB in the mineral mixture may be because the mineral soil contained more available micronutrients compared to mineral free peat. For example, rice has a high demand of silicon and its limitation influences the overall nutrient uptake and plant biomass^58,59^. Thus, detailed nutrient data from after the experiment and nutrient addition experiments would be needed to fully assess the role of nutrients in our observed plant growth response to soil and water treatments.

Ultimately, both water and soil treatments influenced rice plant biomass and allocation strategy, often with opposite effects below and aboveground. Belowground biomass and allocation were halved when mineral soils were added to water-saturated peat. Meanwhile, AGB and total biomass was halved when peat water tables were lowered. These two results suggest that in these rice plants, AGB was driven by water availability while belowground allocation was driven by soil properties. Similar results have been found in studies evaluating AGB and drought response^45^ and root response to soil nutrients^60^ but our study illustrates rice allocation responses to moisture and nutrient conditions in one experimental setting.

### Rice root trait covariation

While traits other than biomass did not have statistically significant responses to the water table treatments, we did see trends of root traits related to increased resource-acquisition strategies responding to water-stressed conditions (e.g. increased SRL; Table S3). We observed slightly higher SRL in the lower water table treatment than in the high water table providing some evidence of increased soil exploration by roots^61^. Root length followed similar trends to BGB (Table S3), but other traits showed no significant responses to water and soil treatments. Some of the lack of responses are also interesting to note. For example, root median diameter was consistently around 2 mm across treatments though the interquartile range was highest in water-saturated mineral soil roots where length and biomass were lowest (Table S3). This suggests a possible trade-off between belowground biomass and allocation to root diameter across our captured trait variation (Figure S5; principal component analysis of all root traits across all treatments). The lack of a response in root diameter to the water treatments is particularly interesting since other studies have reported decreases in root diameter with increases in SRL as a response to drier soil conditions^27,62,63^, but the direction of the response seems to differ between species and growth forms^64^, and rice genotypes^65^. In order to untangle the variation in root diameter and SRL in different water table levels and soil conditions in future studies, it may be worth including measurements of root stele and cortex fractions to fully evaluate nutrient uptake, and water transport and absorption capacity^35,66^.

Another trait trade off emerged between aboveground biomass and belowground allocation for soil exploration. AGB is negatively correlated with root traits such as total root surface area, total root volume, and total root length (Figure S5). Interestingly, this tradeoff disappears in a PCA containing only the high water table trait data. Conversely, this above:belowground tradeoff is pronounced in the low water table trait data (Figure S6), suggesting that, as expected, water stressed conditions may exacerbate plant allocation tradeoffs between aboveground carbon fixation and belowground water (or nutrient) uptake.

Our study provides belowground data from rice plants including trait covariation among rice root traits (Table S3). Even though the root traits show no strong significant responses to treatments, these are valuable data to report given that rice root data are sparse^67^. Lastly, it is also possible that other traits such as root system architecture and maximum rooting depth responded to treatments but were not captured by our methods. Nevertheless, our data add to the limited data on rice root trait covariation and support the notion that rice roots adapt quickly (within a growing season) to water/nutrient stress conditions.

### Relationships between CH_4_ flux and root traits under low water table conditions

So far, a limited number of studies have investigated the effect of root traits on CH_4_ emissions^19^ and little is known about the interactions between rice root traits, methanogens and methanotrophs, and CH_4_ emissions^15,26^. We found some evidence for rice BGB and BGB:AGB predicting CH_4_ emissions, at least in water-stressed conditions (low water table). Our results supported our hypothesis that BGB and CH_4_ flux are positively correlated, likely related to increased BGB facilitating CH_4_ transport through plants and more rhizodeposition potentially increasing substrates for methanogens as well as plant-mediated CH_4_ transport^10,68^. We did not observe the contrary effect of more roots oxygenating the rhizosphere and leading to increased CH_4_ oxidation and reduced net CH_4_ fluxes as seen in paddy soils^22^ and salt marsh ecosystems^69^. There may have been a slight increase in CH_4_ oxidation with increasing root biomass in our study but it may have been overshadowed by increased substrate provision from rhizodeposits and even from a priming effect of root exudates on CH_4_ production^70^, leading to the overall increased CH_4_ emissions. These specific hypotheses remain to be further tested using controlled laboratory incubations.

The lack of a strong relationship between CH_4_ and root traits other than BGB could be due to the use of proxies instead of more direct measurements of CH_4_-related root traits and processes. For example, we assumed root diameter to be a proxy for aerenchyma volume and, therefore, plant-mediated CH_4_ transport from the soil to the atmosphere^19^. However, we found no relationship between root diameter and CH_4_ emissions. One reason could be that we did not measure the distinct and opposing processes of CH_4_ production and consumption, rather we measured the net flux. Similar to the previous discussion on root biomass, increased diameters could result in the confounding effects of increased CH_4_ transport or rhizosphere oxidation^71^ via increased soil reduction–oxidation potential^72,73^, and, ultimately, aerobic CH_4_ consumption. Depending on the local soil conditions and microbial composition and abundance, CH_4_ can be oxidized before reaching the root^74^ or within the root itself^75^, resulting in a decrease in CH_4_ emission. Therefore, it is possible that a lack of relationship between root diameter and net CH_4_ emissions, especially in low water table levels in our study may be because of the confounding processes of methanogenesis (increasing due to increased BGB and root exudation) and methanotrophy (increasing due to large diameter) which cannot be reliably separated based on only net CH_4_ flux. Therefore, we recommend including measurements of rhizosphere oxidation, such as O_2_ concentration from planar optode technology^76^ and redox and O_2_ electrodes^77,78^, in future studies investigating the relationships between BGB and CH_4_ emission. In addition, since root diameter and surface area were used as proxies for CH_4_ transport and root exudation in our study, respectively, it is possible that other more direct measurements of these processes, such as root porosity^22^ and root exudation^70^, could have been better predictors of CH_4_ flux. BGB is also a general proxy for CH_4_ production, consumption and transport and thus overall our weak CH_4_-trait relationships could further indicate that we did not measure root traits potentially more relevant for CH_4_^19^. Nevertheless, since root trait-CH_4_ connections have rarely been investigated, this study is one of the first ones to test the use of different proxies for root-mediated CH_4_ processes in peat soil, and despite the non-significant relationships, these results should motivate researchers into investigating additional CH_4_-relevant root traits.

Another reason for the lack of relationship between root traits and CH_4_ flux could be that the changes in soil properties (i.e. mineral vs. peat) and water table level could have overridden the effects of individual root traits on the net CH_4_ flux. It has been shown in multiple wetland studies that changes in water table and other abiotic variables can have a stronger effect on net soil CH_4_ flux than vegetation^79,80^. However, these relationships have not been adequately investigated in combination with belowground plant traits. Thus, the significant interaction term in the best fit model (Table 1) including low water table and BGB could indicate that root influence on the net CH_4_ flux becomes more relevant only when water table level is decreased, further possibly confirming the overriding effect of the water table. The overriding effect of water table on trait-CH_4_ relationships is also supported when we visualize trait-CH_4_ relationships in the low water table data (PCA in Figure S7). This exploratory analysis suggests that in the tradeoff between plant aboveground and belowground allocation under water stressed conditions (Figure S6a), CH_4_ emissions align with the belowground allocation traits (Figure S7), i.e. if water stressed rice plant allocate more belowground, this could mean an increase in CH_4_ emissions. Future studies should therefore experimentally test the tradeoffs among rice aboveground allocation, belowground allocation and CH_4_ emissions in these systems.

### Implications for Swiss peatlands

Switzerland contains about 28,000 ha of peatland^81^ which represents 1% of the total country area and 2% of the agricultural land^12^. Many of Switzerland’s largest degraded peatlands occur in flat valley bottoms where horticultural and staple crop production dominates. In addition to their high greenhouse gas emissions, these surfaces also require drainage systems to be renewed for the continued cultivation of dry crops, a very costly measure. A wet crop system, such as rice, might provide an alternative agricultural use of these degraded peatland systems. A growing network of rice cultivators has been established in the last years^32^ and increasing temperatures in Switzerland and the associated lengthening of the growing season would further favor rice cultivation. While it is already established that lower water tables and the addition of mineral soils can reduce rice CH_4_ emissions^13^, our results suggest that even under low moisture conditions, further managing or genetically modifying root traits to lower root biomass, albeit with caution^82,83^, could potentially lower CH_4_ emissions. Given our small number of samples and experimental system, this notion would require extensive additional testing.

## Conclusions

We investigated the effects of soil treatment (peat vs., mineral-covered peat) and water level (high vs low) on rarely-studied root traits (biomass, allocation, diameter, length, volume, and surface area) in rice and established previously-untested links among root traits and CH_4_ emission. We found a positive relationship between root biomass and CH_4_ emissions in low water conditions, and the lowest belowground allocation in mineral soil-covered peat soils; thus providing preliminary insights into the potential of minimizing root biomass as a CH_4_ reducing strategy in these systems. This work opens new research directions to understand whether optimizing (and minimizing) BGB and BGB:AGB could be a viable tool for lowering rice CH_4_ emissions from rewetted peatlands.

The results of this study should be understood and concluded with discretion for a few reasons. First, as already discussed above, additional soil nutrient information and measurement of more direct traits would aid in interpreting these results. Secondly, the experiment was short term, representing only one rice species, and using growing season aggregates of both trait and CH_4_ flux data. A longer-term study with time-resolved measurements would allow for better delineation of root trait effects on CH_4_.

Nevertheless, this study provided first insights into how soil type and water affect rice biomass allocation and their link to CH_4_ emission in degraded peatland soils. Degraded peatlands are a widespread ecosystem where rewetting could have high returns for the global carbon cycle-climate feedback^5,84^. In the case of wet rice cultivation, high climate benefits may be accompanied by high economic returns and the continued use of this land for agricultural production, two important socio-economic factors for farmers when determining the management of land.

### Supplementary Information

Supplementary Information 1.

Supplementary Information 2.

## Data Availability

All data used in this study are provided as supplementary materials (Supplementary Information 2). Previously published raw methane data are also available at https://data.mendeley.com/datasets/fxmnty8zf8/1 (Wüst, Chloé; Heller, Sandra; Ammann, Christof; Paul, Sonja; Doetterl, Sebastian; Leifeld, Jens (2023), “CH4 and N2O flux data from wet rice grown on organic soil in Switzerland ”, Mendeley Data, V1, 10.17632/fxmnty8zf8.1). Additional details can also be found in Heller, Sandra. 2021. “Wet rice on organic soils”. *Master Thesis, ETH Zürich*.
