# Supplementary material for: Belowground plant allocation regulates rice methane emissions from degraded peat soils
Source: Sci Rep. 2024 Jun 25;14:14593. doi: 10.1038/s41598-024-64616-1 (PMC11199496; doi:10.1038/s41598-024-64616-1)
Supplement: Supplementary file 1 — Supplementary Information 1. [file 41598_2024_64616_MOESM1_ESM.docx]

# Supplementary Materials

**Belowground plant allocation regulates rice methane emissions from degraded peat soils**

Nijanthini Sriskandarajah^1^, Chloé Wüst-Galley^2^, Sandra Heller^2^, Jens Leifeld^2^, Tiia Määttä^1^, Zutao Ouyang^3^, Benjamin R.K. Runkle^4^, Marcus Schiedung^5,6^, Michael W. I. Schmidt^1^, Shersingh Joseph Tumber-Dávila^7,8^, Avni Malhotra^1,9*^

^1^Department of Geography, University of Zurich, 8057 Zurich, Switzerland

^2^Climate and Agriculture Group, Agroscope, Switzerland

^3^College of Forestry, Wildlife and Environment, Auburn University, Auburn, AL, 36849, USA

^4^Biological & Agricultural Engineering, University of Arkansas, Fayetteville, AR 72701 USA

^5^Department of Environmental Systems Science, ETH Zurich, 8092 Zürich, Switzerland

^6^Thünen Institute of Climate-Smart Agriculture, Bundesallee 68, 38116 Braunschweig, Germany

^7^Department of Environmental Studies, Dartmouth College, Hanover, NH 03755 USA

^8^Harvard Forest, Harvard University, Petersham, MA 01366 USA

^9^Biological Sciences Division, Pacific Northwest National Laboratory, Richland WA 99852 USA

*corresponding author avni.malhotra@pnnl.gov

## Experimental set up of water level regulator


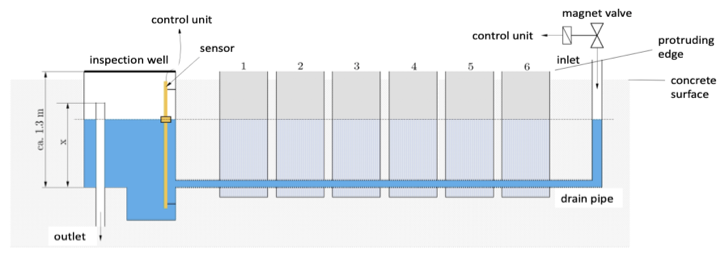


#### Figure S1: Water table of our experimental plots was controlled by several inspection wells. A drain runs from the inspection well through the eight plots in each row, connecting them hydrologically. Figure credit: Agroscope

## Root trait quantification using Rhizovision software

Rhizovision Explorer v2.0.3 (Seethepalli et al. 2021) was used to estimate the root traits of washed roots. Prior to the trait analysis, images were pre-processed to reduce noise from non-root regions of the scans (such as shadows and other blemishes occurring in the image; Figure S2a). To further avoid noise from the edge of the image, the images were cut off by 1.5 cm from each border. Two separate image pre-processing thresholds were selected within the Rhizovision software to more accurately identify finer and thicker roots. For the fine roots, the threshold value was 110 (Figure S2b) and the thicker roots had a value of 95 (Figure S2a). For data analysis, the average of the calculated values of both thresholds was taken. All images were analyzed in this way. From the image analysis output, the following root traits were computed: total root length for each of the 24 root systems, average root diameter, maximum root diameter, minimum root diameter, total root length in different diameter classes between 1-6 mm, total root surface area, and total root volume. Rhizovision estimates total root length as the sum of the Euclidean distances among the skeletal pixels, and average diameter represents the distance of the skeletal pixel to radius, which is then doubled. The total root volume and total surface area was calculated using the radii of each skeletal pixel and the length of the roots [^76^](https://paperpile.com/c/RO0ri7/1f6EX). The entire scan (minus the 1.5 cm border) was identified as the region of interest, using the “broken roots” mode to analyze all of the root segments, since the root system was not intact, with the DPI set to 600. During image processing, the filter non-root objects parameter was set to 2, so that non-root objects could be removed. Setting a higher value for non-root (e.g., 4) started to count roots also as a non-root object. The Rhizovision analysis resulted in a csv file of the root trait values across the two thresholds we determined for fine and thick roots.

a.
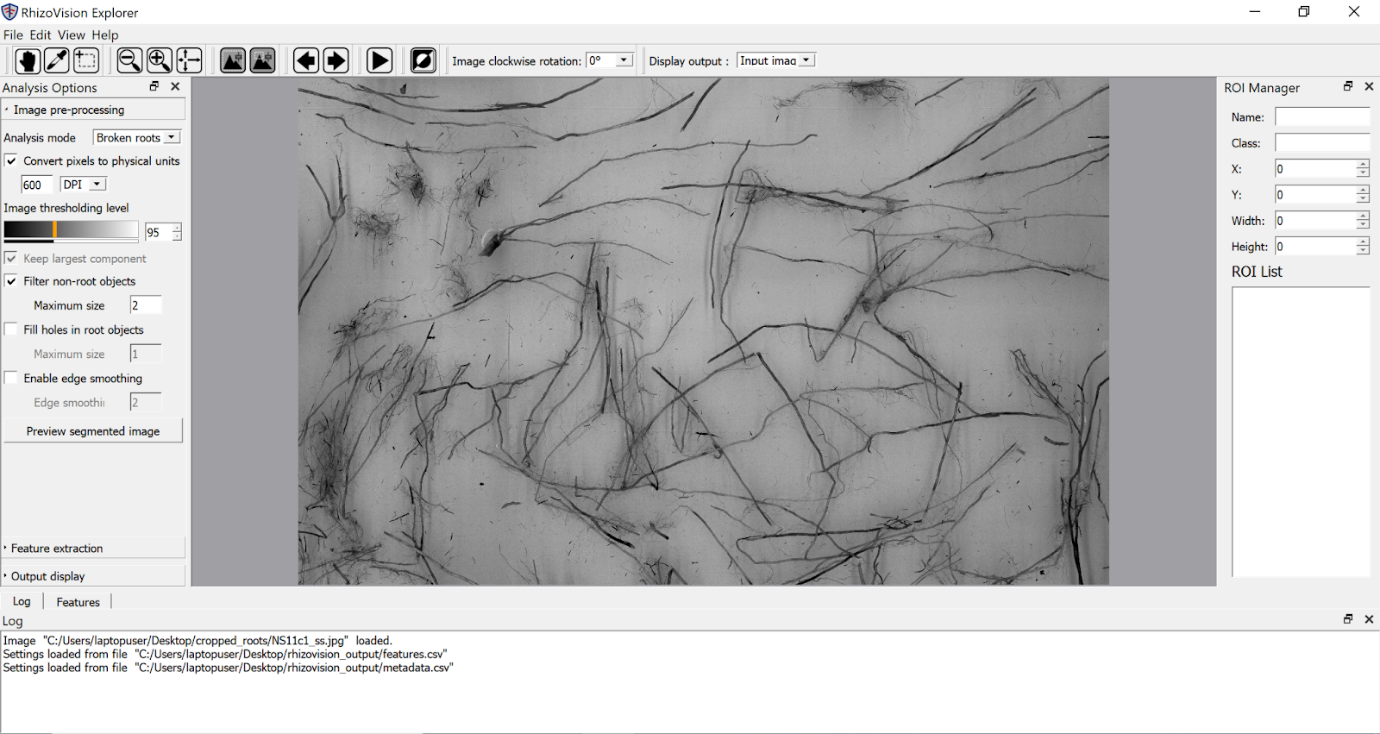


b.


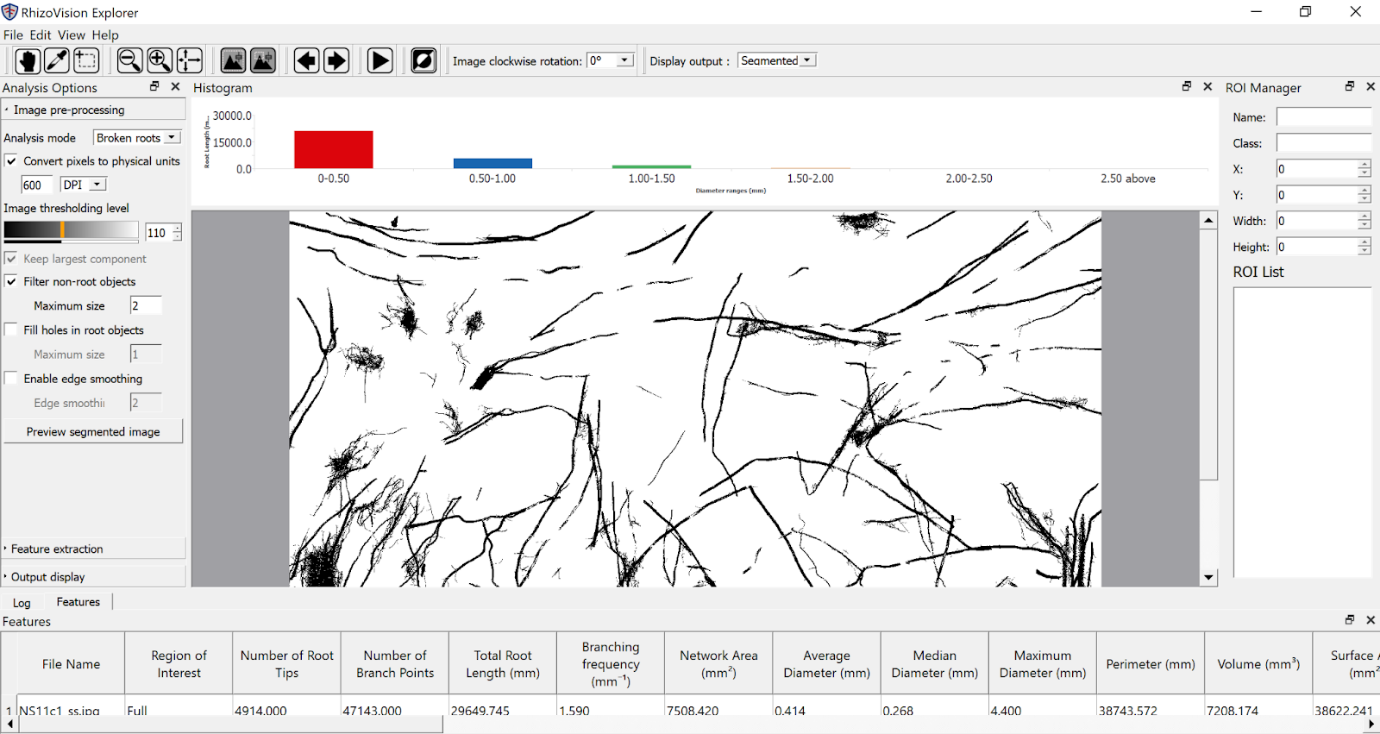


#### Figure S2: Example screenshots of the Rhizovision workflow showing the image pre-processing (a), and the analyzed output (b). a. Rhizovision Explorer window with a greyscale image from a sample demonstrating the image pre-processing, with the analysis mode set to “Broken roots,” the DPI is set to 600, the threshold is set to 95 (for thicker), and the ROI is the entire image. b. Rhizovision Explorer window depicting the segmented output which had the threshold value of 110 (for finer roots), demonstrating the distribution of traits as a figure (barplot above image) and a table of trait values (table below image).

#### Figure S3. Soil nutrient concentrations varied by soil type but not by water table. Notably, C:N was lower in peat than in mineral soil (Wilcoxon test, Z= -3.5, p= 0.0005). C and N concentrations of mineral and peat soils also differed significantly (p= 0.0001 in Wilcoxon tests). Note that one C:N data point for low-mineral and high-mineral is not shown as it is an outlier (values of 300 due to a very low [N]).

#### Figure S4: Raw data on CH_4_ flux and a) BGB, and b) BGB:AGB corresponding to Figure 5.

#### Figure S5. A principal component analysis of all the measured traits (shown as black arrows) in this study provides insights into rice plant trait covariation and trade-offs. The gray points are the 24 measured plots.

#### Figure S6. A principal component analysis of all the measured traits (shown as black arrows) in this study provides insights into rice plant trait covariation and trade-offs in the low and high water table treatments (both soil types are included).

#### Figure S7. A principal component analysis plant traits and CH_4_ in the low water table treatments (both soil types are included).

#### Table S1. Timeline and details of fertilizer (all in kg/ha) application during the experiment. Other important dates are as follows: First gas measurements: 20th May, Seedlings planted out: 26th May, Harvest of rice: 15th to 20th October (depending on treatment), Last gas measurement considered in this study: 20th October. All dates are for the year 2021.

|  | 5th May | 11th May | 19th May | 25th May | 9th June | 13th July | 17th August |
| --- | --- | --- | --- | --- | --- | --- | --- |
| Stage | Seedling | Seedling | Seedling | Seedling | Start of tillering | Mid-tillering | Start of panicle |
| Fertilizer | Wuxal | Wuxal | Wuxal | Wuxal |  |  |  |
| Total N |  |  |  |  | 37 | 37 | 37 |
| Total P |  |  |  |  | 13 | 13 | 0 |
| Total K |  |  |  |  | 60 | 60 | 0 |

#### Table S2. Pairwise comparison outputs for plant components. Each treatment level is compared and p values < 0.1 are highlighted as possible treatments that were significantly different from each other. Note that our study had low sample sizes (n=4 for high-mineral and low-mineral; n=8 for high-peat and low-peat).

| *Trait* | *Treatment pairs* | | *Kruskal Wallis Chi square* | *Kruskal Wallis degrees of freedom* | *Steel-Dwass score mean difference* | *Standard error of difference* | *Z score* | *p-value* |
| --- | --- | --- | --- | --- | --- | --- | --- | --- |
| **BGB** | **Overall model** | | **12.5** | **3** |  |  |  | **0.0058** |
|  | low-peat | high-mineral |  |  | 5.8 | 2.2 | 2.6 | 0.0421 |
|  | high-peat | high-mineral |  |  | 5.3 | 2.1 | 2.6 | 0.0524 |
|  | low-mineral | high-mineral |  |  | 3.8 | 1.7 | 2.2 | 0.1331 |
|  | low-peat | low-mineral |  |  | 3.2 | 2.2 | 1.4 | 0.472 |
|  | low-peat | high-peat |  |  | -1.2 | 2.3 | -0.5 | 0.9541 |
|  | low-mineral | high-peat |  |  | -4.1 | 2.1 | -2.0 | 0.1939 |
| **AGB** | **Overall model** | | **16.2** | **3** |  |  |  | **0.001** |
|  | high-peat | high-mineral |  |  | 0.0 | 2.1 | 0.0 | 1 |
|  | low-peat | low-mineral |  |  | -3.6 | 2.2 | -1.6 | 0.3709 |
|  | low-mineral | high-mineral |  |  | -3.8 | 1.7 | -2.2 | 0.1331 |
|  | low-mineral | high-peat |  |  | -5.3 | 2.1 | -2.6 | 0.0524 |
|  | low-peat | high-mineral |  |  | -5.4 | 2.2 | -2.5 | 0.0659 |
|  | low-peat | high-peat |  |  | -7.1 | 2.3 | -3.1 | 0.0116 |
| **BGB:AGB** | **Overall model** | | **15.3** | **3** |  |  |  | **0.0016** |
|  | low-peat | high-mineral |  |  | 5.8 | 2.2 | 2.6 | 0.0421 |
|  | low-peat | high-peat |  |  | 5.8 | 2.3 | 2.5 | 0.0617 |
|  | high-peat | high-mineral |  |  | 5.3 | 2.1 | 2.6 | 0.0524 |
|  | low-peat | low-mineral |  |  | 4.7 | 2.2 | 2.1 | 0.1457 |
|  | low-mineral | high-mineral |  |  | 3.8 | 1.7 | 2.2 | 0.1331 |
|  | low-mineral | high-peat |  |  | 0.0 | 2.1 | 0.0 | 1 |
| **BGB+AGB** | **Overall model** | | **13.6** | **3** |  |  |  | **0.0035** |
|  | high-peat | high-mineral |  |  | 3.7 | 2.1 | 1.8 | 0.2755 |
|  | low-mineral | high-mineral |  |  | -3.3 | 1.7 | -1.9 | 0.2382 |
|  | low-peat | low-mineral |  |  | -3.9 | 2.2 | -1.8 | 0.2813 |
|  | low-peat | high-mineral |  |  | -4.3 | 2.2 | -2.0 | 0.206 |
|  | low-mineral | high-peat |  |  | -5.3 | 2.1 | -2.6 | 0.0524 |
|  | low-peat | high-peat |  |  | -6.0 | 2.3 | -2.6 | 0.0456 |

#### Table S3: Soil and water treatment response of root traits. Medians and interquartile ranges are reported for each trait. SRL= specific root length. We observed no significant differences among the four treatment types (Kruskal-Wallis tests p>0.05). It is worth noting that high-mineral treatment had roughly half the total root length of the low-peat treatment (Kruskal-Wallis test chi square = 6.2, p=0.0980; Steel-Dwass pairwise comparison Z=2.17 p= 0.13).

|  |  |  | Median | | | |  | Interquartile Range | | | | |
| --- | --- | --- | --- | --- | --- | --- | --- | --- | --- | --- | --- | --- |
| Treatment | n |  | Total root surface area (cm2) | Average root diameter (mm) | Total root volume (cm3) | SRL (m/g) |  | Total root length (m) | Total root surface area (cm2) | Average root diameter (mm) | Total root volume (cm3) | SRL (m/g) |
| low-mineral | 4 |  | 113.05 | 2.00 | 14.59 | 4.77 |  | 37.73 | 83.81 | 0.22 | 12.74 | 5.78 |
| high-mineral | 4 |  | 42.59 | 2.27 | 12.10 | 4.28 |  | 11.71 | 42.66 | 0.78 | 15.99 | 2.63 |
| low-peat | 8 |  | 117.07 | 1.96 | 19.96 | 3.95 |  | 58.17 | 147.78 | 0.36 | 45.17 | 8.45 |
| high-peat | 8 |  | 79.70 | 2.06 | 11.88 | 2.86 |  | 35.21 | 83.72 | 0.25 | 16.20 | 5.80 |

Table S4: Pretreatment soil properties for the peat and mineral soil. While limited, these properties provide some information about possible differences in micronutrients between these different soil types. All units are in cmol per kg soil. Methods for these quantifications are provided in Agroscope ([2020](https://ira.agroscope.ch/de-CH/publication/46182)). Briefly, bases were extracted with a pH 8.1 buffered Barium chloride/Triethanolamine (TEOA) solution. Calcium, Magnesium, Sodium and Potassium were quantified using an atomic absorption spectrometer.

|  | Mineral soil | Degraded peat soil |
| --- | --- | --- |
| Calcium | 4.74 | 67.95 |
| Potassium | 0.31 | 0.2 |
| Magnesium | 0.28 | 7.94 |
| Sodium | 0.03 | 0.12 |
| Cation exchange capacity | 5.38 | 106.61 |
